# Unusual gastric band migration outcome: distal small bowel obstruction and coming out per-rectum

**Published:** 2012-11-20

**Authors:** Alkhalifah Bassam

**Affiliations:** 1 PSRMMC, Riyadh, Saudi Arabia

**Keywords:** Laparoscopic adjustable gastric band, bariatric surgery, band migration, small bowel obstruction, access-port infection

## Abstract

We describe a case of unusual gastric band migration outcome. A 54 years old female was submitted to laparoscopic adjustable gastric band in September 2001. In September 2009 she developed access-port infection which needed drainage and access-port removal. Three months later in December 2009 the patient was investigated due to abdominal pain and abdominal distention. X-rays and Abdomen CT revealed migrated gastric band which is completely intraluminal with its connecting tube, causing transient distal small bowel obstruction and subsequently comes out per-rectum. Band erosion and intragastric migration is a late complication that frequently needs surgical removal. There are few reported cases in the literature of migrated gastric band removal by endoscopy. However according to my knowledge, this is the first reported case of migrated gastric band coming out per rectum without need for surgical or enoscoipic removal.

## Introduction

Laparoscopic adjustable gastric band (LAGB) is the most frequent bariatric surgery performed worldwide [1]. It is an effective treatment of morbid obesity with low morbidity and mortality rates. The overall long term complication rates ranging from 6 to 26% [2, 3]. They can be band or port related complications. Band related complications include slippage, gastric perforation and bleeding, pouch dilatation, esophageal dilatation and dysmotility, food intolerance and intragastric migration (erosion). Port-associated complications include infection, disconnection, leakage or migration.

## Patient and observation

A 54 years old female patient (weight 108 kg height 162 cm, body mass index (BMI) = 41.2 kg/cm^2^) was submitted for laparoscopic adjustable gastric band in September 2001. Her comorbidities include non-insulin dependent diabetes and hypertension. The post-operative course reported to be straightforward with no immediate complications. However she started to be followed up in other hospital with no available documented data about reduction in weight in the few years after the surgery.

**Figure 1:**
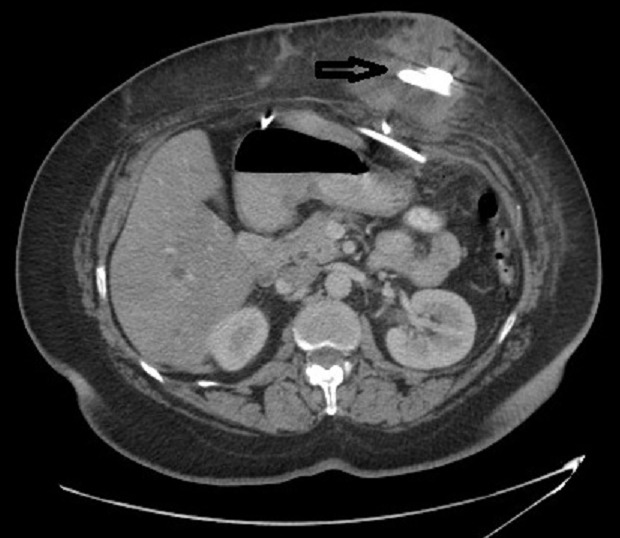
Abdomen CT scan showed fluid collection around the access-port (arrow)

At September 2009, she presented to the emergency department complaining of fever, periumblical pain and swelling. On examination there is superficial periumblical swelling and tenderness. Blood tests revealed leukocytosis. Abdominal contrast enhanced CT scan showed 6.5 X 4.5cm fluid collection surrounding the access port (Figure 1). Under general anesthesia, incisional drainage and access-port removal was performed. The remaining part of connecting tube left in the abdominal cavity. The patient covered with antibiotic regimen and released from the hospital few days later doing well.

**Figure 2:**
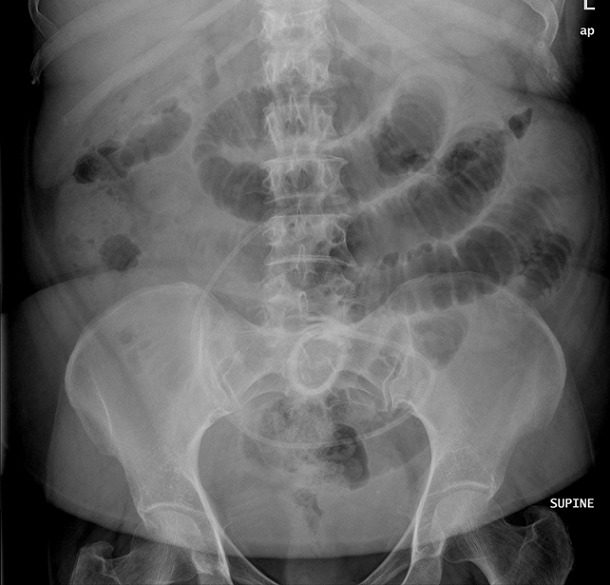
Supine abdomen X-Ray showed small bowel dilatation and ectopic lower abdominal position of gastric band.

**Figure 3:**
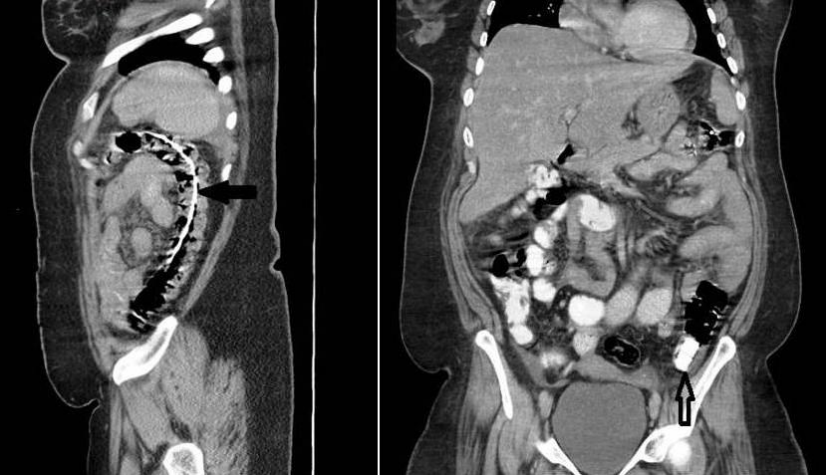
Abdomen sagittal and coronal CT scan showed gastric band connecting tube at splenic flexure and descending colon (thick arrow), the gastric band seen in the distal descending colon (open arrow).

**Figure 4:**
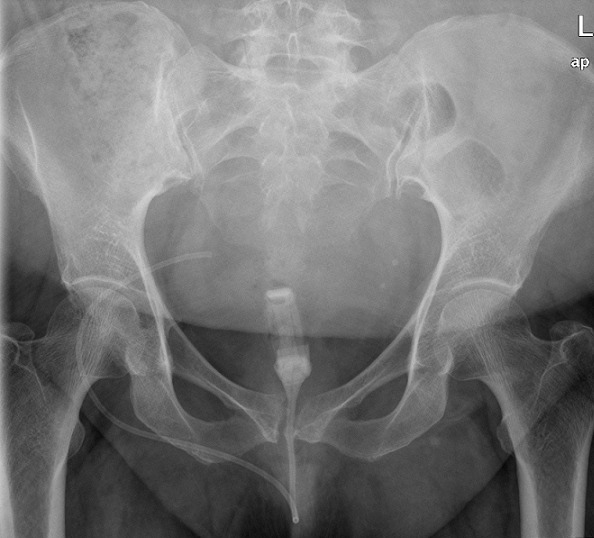
Pelvic X-RAY hours after the CT showed the gastric band and its connecting tube in the lower pelvis

Three months later, the patient presented again to the emergency department with colicky abdominal pain and abdominal distention. Supine and erect abdomen x-ray revealed small bowel dilatation and multiple air-fluid levels (Figure 2). Interestingly, the gastric band and its connecting tube seen in the lower abdomen at midline probably in the distal ilial loops (Figure 2). The patient was given oral contrast (Gastrografin^®^) and contrast enhanced abdomen CT scan performed later. It showed resolution of bowel dilatation with the band and its connecting tube noted at transverse / descending colon (Figure 3). Few hours later the patient started to have rectal pain and X-ray requested. It showed that the gastric band at rectal area (Figure 4). Per rectal examination performed and the gastric band removed with no difficulties. Repeated abdominal X-Ray confirms complete removal of gastric band and its connecting tube. The patient symptoms relieved and she was discharged in good condition. The patient followed up in the surgery outpatient clinic and she was doing fine with no GIT symptoms.

## Discussion

Laparoscopic adjustable gastric band is the least invasive bariatric surgery, which has the advantage of preserving the anatomy of gastrointestinal tract [6]. Access port infection usually requires disconnection and removal of the access port and placement of the tube in abdominal cavity and after a local healing, the tube can be reconnected to a new access port laparoscopically [4, 5]. Infections of the access-port usually occur due to inadequate aseptic technique during the puncture of the port. Therefore strict asepsis while accessing the port is necessary to prevent such complication.

There are few reported cases in the literature of unusual gastric bands migration outcome like intra colic penetration [8, 9] and small bowel obstruction [10], with several reported cases of endoscopic removal of migrated gastric band [11]. I present a case of migrated gastric band coming out per rectum which is according to my knowledge, never reported migrated gastric band outcome.

Band migration is a major complication of LAGB that can be due to early or late erosion. Early erosion thought to be related gastric wall damage during the initial operation. Late erosion takes place over a long period of time; usually after two years. It is the result of a destructive process and an effective self-healing of the gastric wall [7, 8]. Late band erosion is often asymptomatic or only associated with non-specific symptoms, such as weight regain, unspecific epigastric pain, vomiting, or port-associated infection .There are also more serious signs like acute upper gastrointestinal bleeding and bowel obstruction [1, 6, 8]. Upon diagnosis, the eroded band should be removed. There is no immediate need for band removal if patient is, for the most part, asymptomatic. A complete band migration can be awaited allowing for endoscopic retrieval rather than laparoscopic removal [1, 7, 8]. However if the port was removed and the gastric band is left intraabdominal, bowel perforation [8, 9] or complete intra gastric migration with the remaining connecting tube can occur and subsequent passage of the band to the bowel distally, causing obstruction or even coming out per rectum as in our case.

## Conclusion

Access-Port infection is frequently seen as complication of inadequate asepsis during puncturing the port. It is usually necessitates disconnection and removal of the port. It is important to exclude any associated gastric band erosion since most of the patients are asymptomatic. If the migrated band left with the tube not connected to the port, it can leads to further erosion of stomach or bowel [8, 9]. Furthermore if the migrated band was not removed laprascopically or endoscopically, it can pass distally causing bowel obstruction that needs surgical intervention. However in our case the band passed whole the way causing transient obstruction and subsequently comes out per rectum with no subsequent events.
